# Supplementary figures and images for: A single N-terminal amino acid determines the distinct roles of histones H3 and H3.3 in the Drosophila male germline stem cell lineage
Source: PLoS Biol. 2023 May 1;21(5):e3002098. doi: 10.1371/journal.pbio.3002098 (PMC10174566; doi:10.1371/journal.pbio.3002098)

S1 Fig:

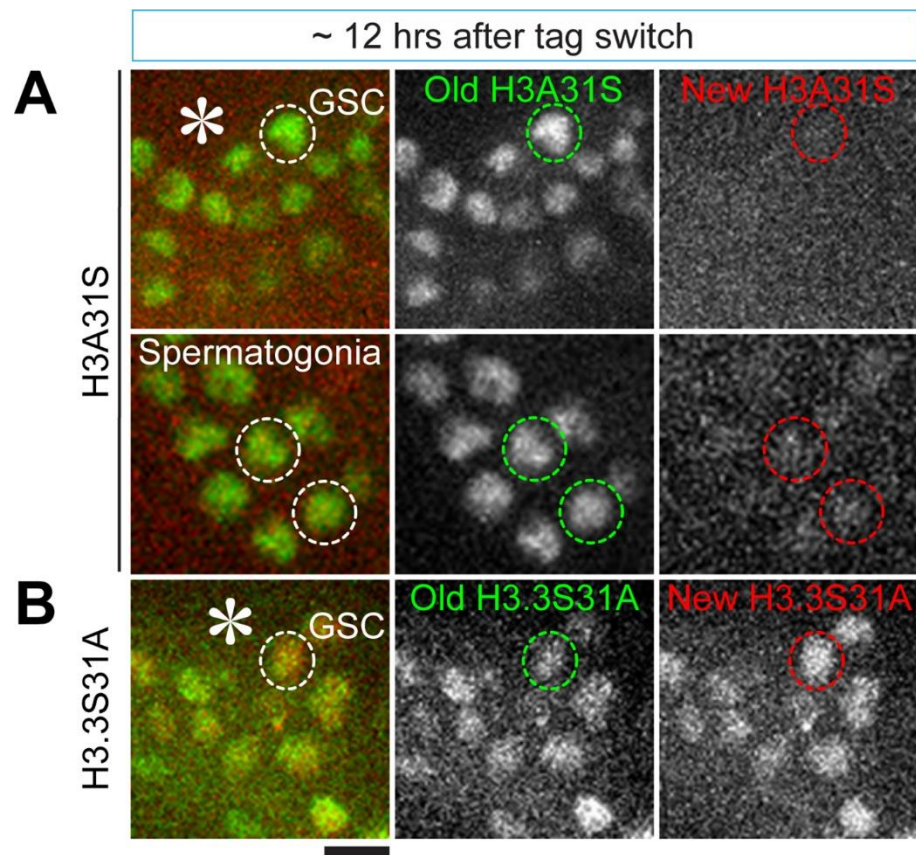

Supplement: S1 Fig — (A) Testis tip of the nos>FRT-H3A31S-eGFP-FRT- H3A31S-mCherry and (B) nos>FRT-H3.3S31A-eGFP-FRT- H3.3S31A-mCherry transgenic lines. Both old histone (eGFP labeled) and new histone (mCherry labeled) signals are shown in GSCs and SGs, 12 hours after heat shock–induced tag switch. Given the cell cycle length for GSCs (12–16 hours) and SGs (10–12 hours) [54], 12 hours post-heat shock recovery time should allow germ cells to be within or just at the accomplishment of the first cell cycle. There were undetectable or low levels of new H3A31S (A), but abundant new H3.3S31A (B). These cell cycle dependence of each mutant histone is similar to their corresponding incorporation mode as WT H3 is S phase dependent and WT H3.3 is S phase independent, consistent with previous reports [68,29]. GSC, germline stem cell; SG, spermatogonial cell; WT, wild-type. (PDF) [file pbio.3002098.s001.pdf]

S2 Fig:

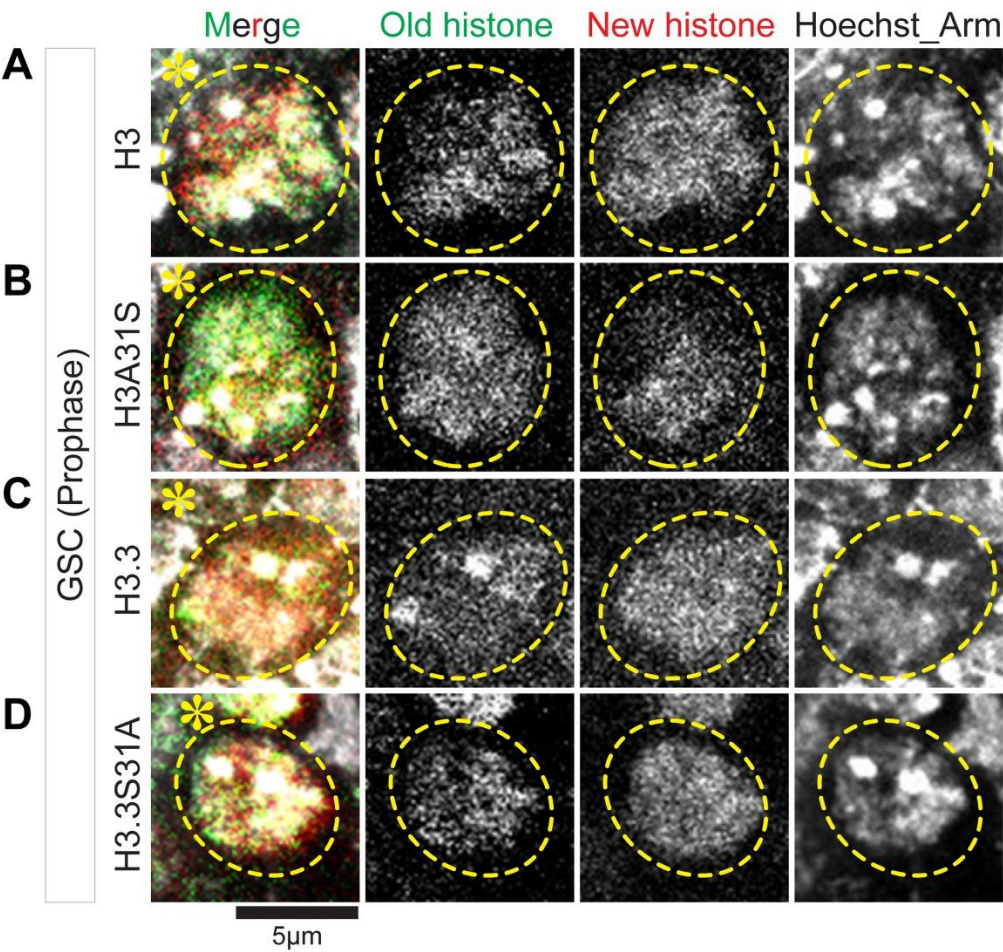

Supplement: S2 Fig — (A-D) Old versus new control H3 (A), mutant H3A31S (B), control H3.3 (C), mutant H3.3S31A (D) distribution in mitotic (prophase) GSCs labeled with Hoechst and Armadillo (white, DNA marker and Hub marker, respectively), showing old (green) and new (red) histone distribution patterns. Asterisk: niche. Scale bars: 5 μm. GSC, germline stem cell. (PDF) [file pbio.3002098.s002.pdf]

S3 Fig:

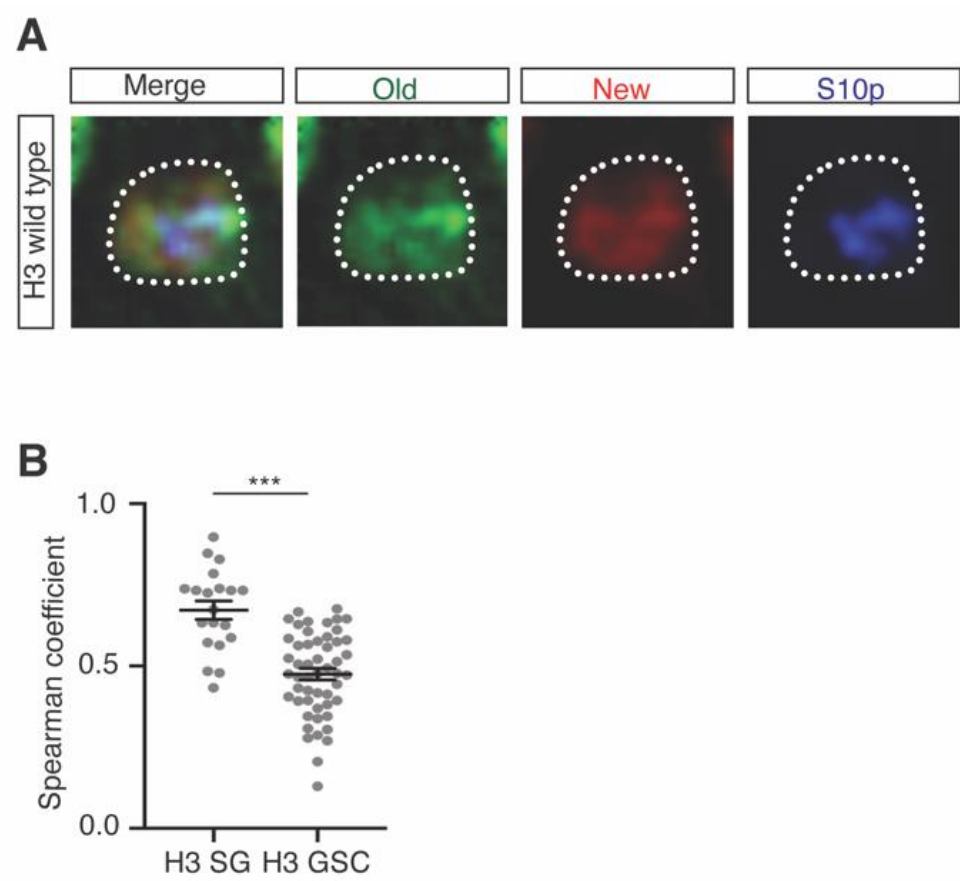

Supplement: S3 Fig — (A) Old versus new WT H3 in an SG. (B) Quantification colocalization patterns of H3 WT in SGs versus GSCs show significant differences. Unpaired t test to compare the 2 individual datasets to each other. *** P < 0.001. The data underlying this panel can be found in S8 Table. GSC, germline stem cell; SG, spermatogonial cell; WT, wild-type. (PDF) [file pbio.3002098.s003.pdf]

**S4 Fig:**

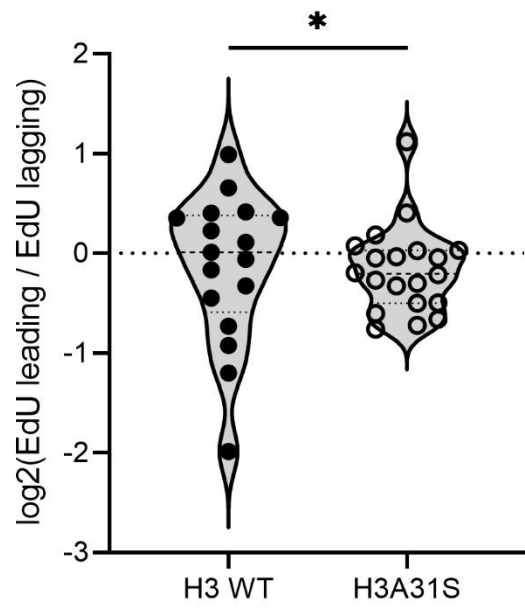

Supplement: S4 Fig — Quantification of EdU distribution on H3A31S-labeled chromatin fibers shows significant differences from EdU distribution on WT H3-labeled chromatin fibers. The EdU asymmetry is less on H3A31S-labeled chromatin fibers than on WT H3-labeled chromatin fibers. * P < 0.05 using F test to compare variances. The data underlying this panel can be found in S9 Table. WT, wild-type. (PDF) [file pbio.3002098.s004.pdf]

S5 Fig:

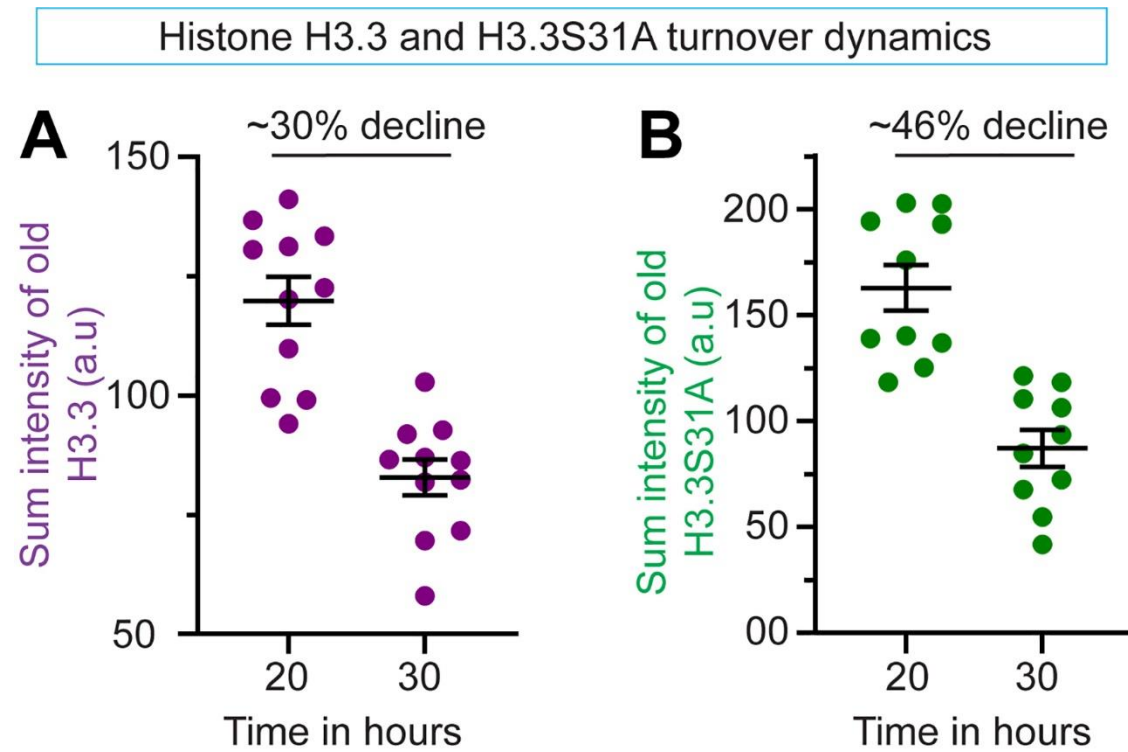

Supplement: S5 Fig — The time-course recovery experiment was performed using live cell imaging (at 20 hours and 30 hours) after heat shock to examine old histones turnover in GSCs. The 3D quantification of old histone (sum intensity) was performed in GSCs at each time point after heat shock recovery. Individual data points are representative of individual GSCs at each time point. (A) H3.3 (20 hours: n = 11, 1198965.5 ± 50341.5; 30 hours: n = 11, 828469.4 ± 37435.7) and (B) H3.3S31A (20 hours: n = 10, 1629018.0 ± 107460.6; 30 hours: n = 10, 872505.4 ± 86988.8). Error bars represent the SEM. The data underlying these panels can be found in S10 Table. GSC, germline stem cell; SEM, standard error of the mean. (PDF) [file pbio.3002098.s005.pdf]

**S6 Fig:**

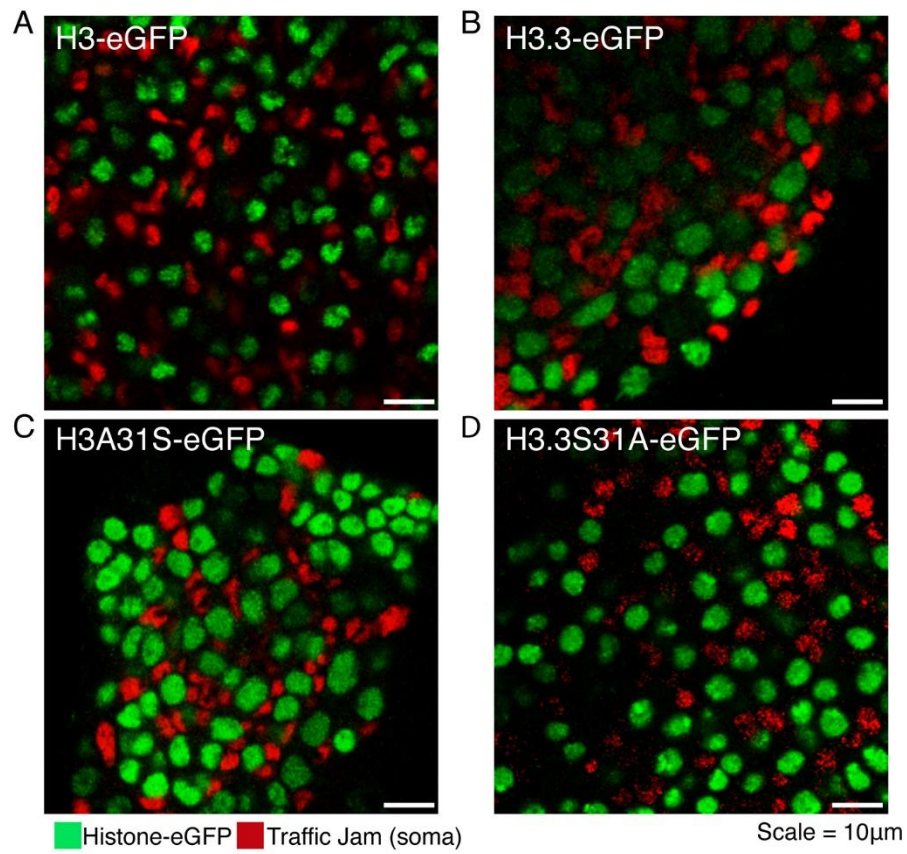

Supplement: S6 Fig — Coexpression of the unpaired and eGFP-tagged histone transgenes with the nanos promoter produces germ cell and somatic cell tumors where histone-eGFP is expressed only in germ cells. Each of the tagged histones (green) are shown in complementary with somatic cyst cells stained with Traffic Jam (red). Scale bar: 10 μm. (PDF) [file pbio.3002098.s006.pdf]

**S7 Fig:**

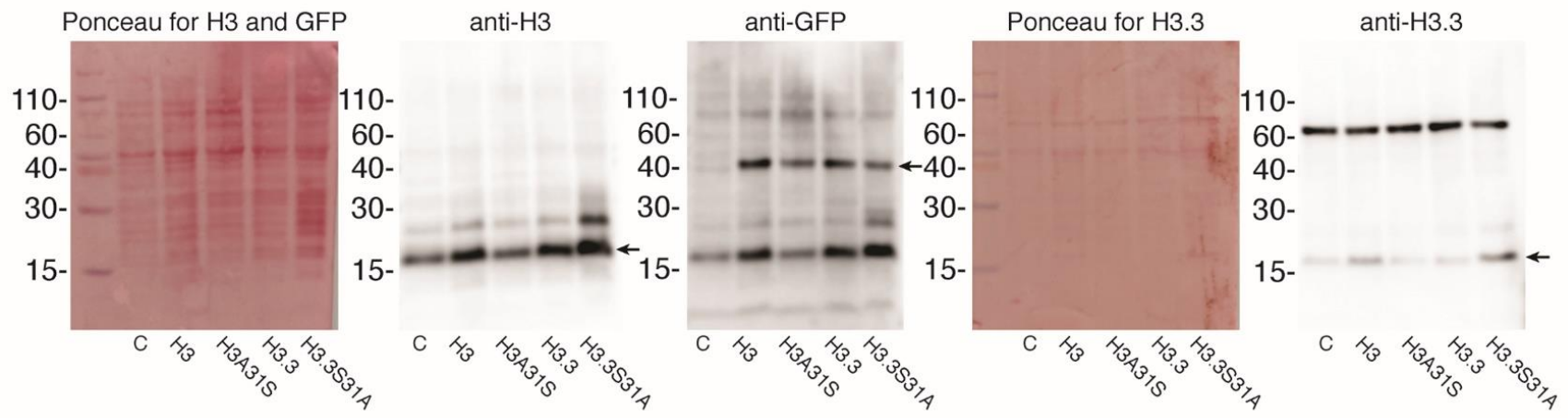

Supplement: S7 Fig — Endogenous and transgenic histones H3 and H3.3 were blotted for in control (C) nos> Upd and nos> Upd; histone-GFP testes. Endogenous histone H3 and H3.3 (approximately 15 kDa, arrows in respective blots) can be seen in addition to transgenic GFP-tagged histones (approximately 40 kDa, arrow in anti-GFP blot). Ponceau staining of each membrane shows total protein loaded. (PDF) [file pbio.3002098.s007.pdf]

**S8 Fig:**

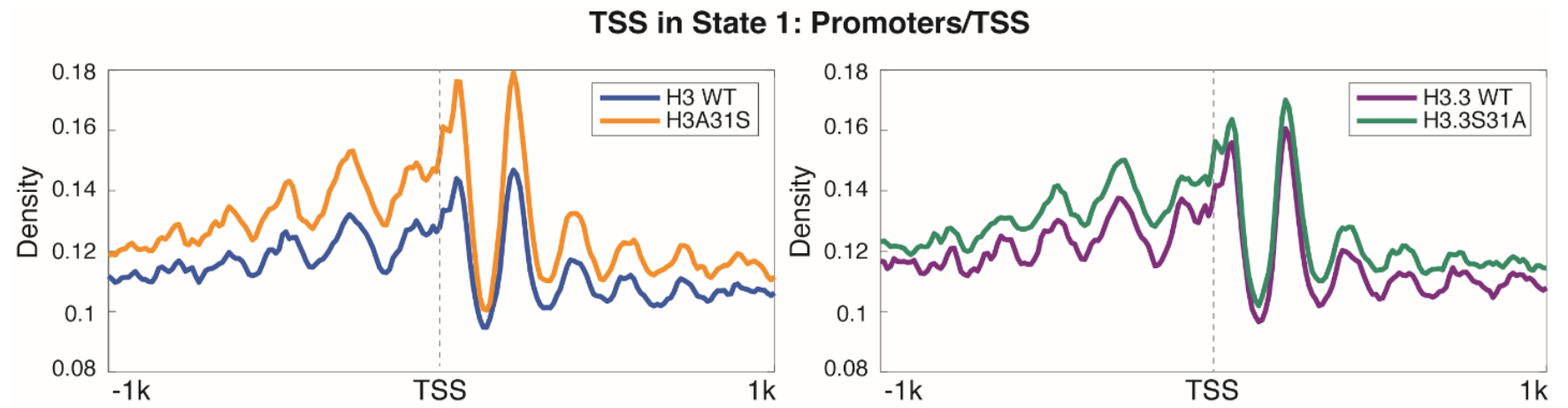

Supplement: S8 Fig — Unlike histones H3 and H3A31S (left), the average density plot of histone H3.3 and its mutant H3.3S31A (right) show no differences in occupancy at TSS extracted from chromatin state 1. TSS, transcription start site. (PDF) [file pbio.3002098.s008.pdf]
