# Supplementary material for: A single N-terminal amino acid determines the distinct roles of histones H3 and H3.3 in the Drosophila male germline stem cell lineage
Source: PLoS Biol. 2023 May 1;21(5):e3002098. doi: 10.1371/journal.pbio.3002098 (PMC10174566; doi:10.1371/journal.pbio.3002098)
Supplement: S1 Table — (PDF) [file pbio.3002098.s009.pdf]

S1 Table:

	Number of GSC		Tumor Present (N/Y)		Hub Size (μm^2)	
	**H3 WT**	**H3A31S**	**H3 WT**	**H3A31S**	**H3 WT**	**H3A31S**
1	9	10	N	Y	72.22	154.984
2	7	10	N	Y	72.336	336.012
3	6	10	N	Y	75.592	106.833
4	8	10	N	Y	71.522	199.133
5	7	10	N	Y	91.124	243.993
6	9	10	N	Y	61.714	242.899
7	6	10	N	Y	106.786	227.034
8	8	10	N	Y	89.574	560.199
9	10	10	N	Y	80.373	331.096
10	7	10	N	N	98.154	193.89
11	6	11	N	N	82.803	687.417
12	8	12	N	N	103.32	290.353
13	10	13	N	N	100.4332	563.578
14	10	14	N	N	115.23	225.401
15	9	14	N	N	170.32	199.598
16	6	15	N	N	80.34	112.701
17	9	16	N	N	96.324	237.138
18	11	17	N	N	98.34	596.783
19	9	16	N	N	130.3	596.783
20	8	18	N	N	93.43	86.175
21	9	18	N	N	90.2343	136.415
22	10	18	N	N	112.34	207.937
23	8	19	N	N	107.34	108.388
24	10	20	N	N	104.23	81.562

25	10	20	N	N	80.32	119.811
26	10	21	N	N	85.34	185.957
27	10	22	N	N	70.23	103.374
28	10	22	N	N	88.454	229.801
29	9	22	N	N	90.234	108.45
30	8	18	N	N	89.35	267.98
