# Supplementary material for: A single N-terminal amino acid determines the distinct roles of histones H3 and H3.3 in the Drosophila male germline stem cell lineage
Source: PLoS Biol. 2023 May 1;21(5):e3002098. doi: 10.1371/journal.pbio.3002098 (PMC10174566; doi:10.1371/journal.pbio.3002098)
Supplement: S2 Table — (PDF) [file pbio.3002098.s010.pdf]

S2 Table:

For Fig 2B:

	**1 DAY**		**5 DAY**		**10 DAY**	
	**H3.3 WT**	**H3.3S31A**	**H3.3 WT**	**H3.3S31A**	**H3.3 WT**	**H3.3S31A**
1	12	10	10	8	10	10
2	10	10	12	8	9	9
3	14	10	10	9	9	9
4	13	10	12	10	9	9
5	10	9	15	11	9	8
6	10	10	13	7	9	8
7	10	9	10	6	9	8
8	10	10	8	9	10	7
9	10	11	9	8	10	7
10	9	12	10	10	8	7
11	8	12	12	8	8	5
12	9	10	13	9	8	5
13	9	9	12	6	8	5
14	9	11	15	7	11	5
15	10	11	13	7	7	6
16	10	11	10	7	7	6
17	11	11	10	9	8	6
18	11	12	10	10	8	7
19	12	11	10	10	8	7
20	14	12	11	10	10	7
21	14	12	9	6	10	8
22	10	10	10	10	10	8
23	10	10	12	8		8

24	10	12	11	8		9
25	9	12	8	8		9
26	10	12	7	9		9
27	12	11	10	9		9
28	12	14	11	8		
29	10	9	8	10		
30	11	9	9	10		

For Fig 2C:

	H3.3 WT	H3.3S31A	H3.3 WT	H3.3S31A	H3.3 WT	H3.3S31A	H3.3 WT	H3.3S31A
1	113	89	86	143	88	82	157	93
2	130	149	157	135	103	58	162	116
3	124	158	154	141	81	77	157	114
4	121	153	153	141	136	130	112	129
5	135	176	153	127	117	108	75	110
6	151	161	154	137	107	113	126	86
7	138	149	146	132	75	60	92	119
8	163	143	164	138	119	55	159	112
9	133	161	152	137	104	70	96	75
10	167	137	179	161	79	50	142	40
11	164	151	155	146	102	70		17
12	116	164	113	92				
13	63	87	99	122				
14	88	93	77	76				
15		60						

For Fig 2E:

	H3	H3.3S31A
1	59881	28769
2	78967	19985
3	32511	7946
4	56733	7503
5	163448	18940
6	132532	21816
7	66852	35025
8	50997	47261
9	64667	23953
10	136280	27869
11	187285	-6870
12	135102	24899
13	188863	3166
14	54171	16402
15	48664	87501
16	44977	51608
17	47493	25982
18	40704	69806
19	55233	64683
20	76958	115184
21	143428	508
22	168891	13372
23	154756	40281
24	54936	51094
25	72554	21205

26	70797	38302
27	57380	45340
28	40279	29492
29	64756	18817
30	106730	29054
31	84809	32523
32	92251	72083
33	49942	18965
34	93608	35999
35	93595	7688
36	88839	73241
37	123463	3264
38	57254	78916
39	47667	21117
40	67542	2232
41	88735	6210
42	46997	4203
43	92333	10058
44	96380	15735
45	22848	3041
46	21911	60898
47	11980	22080
48	10064	23448
49	339	59483
50	9989	
51	4336	
52	3640	

53	11177	
54	2757	
