# Supplementary material for: A single N-terminal amino acid determines the distinct roles of histones H3 and H3.3 in the Drosophila male germline stem cell lineage
Source: PLoS Biol. 2023 May 1;21(5):e3002098. doi: 10.1371/journal.pbio.3002098 (PMC10174566; doi:10.1371/journal.pbio.3002098)
Supplement: S3 Table — (PDF) [file pbio.3002098.s011.pdf]

S3 Table:

Quantification of histone inheritance pattern in asymmetrically dividing GSCs or symmetrically dividing SGs, which express the corresponding wild-type or mutant histones. Table showing log_2 ratios of the corresponding total histones between sister chromatids in telophase cells.

#	Old H3 (GSC)	New H3 (GSC)	Old H3 (SG)	New H3 (SG)	Old H3A31S (GSC)	New H3A31S (GSC)	Old H3.3 (GSC)	New H3.3 (GSC)	Old H3.3S31A (GSC)	New H3.3S31A (GSC)
1	1.585	0.529	0.108	0.205	-0.060	-0.471	0.676	0.301	-0.102	0.356
2	1.084	0.424	0.085	0.136	0.188	0.300	0.626	0.218	0.128	0.190
3	1.067	0.558	0.174	0.120	0.094	0.144	0.642	0.356	0.121	0.371
4	0.775	0.269	0.041	0.017	0.464	0.392	0.120	-0.054	0.527	0.147
5	0.938	0.437	0.017	0.172	0.137	0.083	0.299	0.220	0.461	0.825
6	0.983	0.526	0.056	0.069	0.494	0.175	-0.357	-0.283	0.093	0.401
7	1.084	0.427	0.136	0.184	0.344	0.385	-0.122	-0.687	-0.161	0.625
8	0.938	0.437	0.324	0.068	0.446	0.238	0.040	0.169	0.383	0.335
9	0.983	0.526	0.194	0.116	0.286	-0.293	0.214	0.203	-0.162	-0.302
10	0.595	0.276	0.000	0.160	0.464	0.376	0.176	0.113	0.472	0.238
11	0.641	0.592	0.144	0.076	0.237	-0.349	-0.456	-0.231	0.154	0.165
12	0.777	0.224	0.076	0.001	0.048	0.421			0.161	0.184
13	1.267	0.077	0.175	0.140	-0.226	-0.025			-0.026	-0.150
14	1.006	0.578	0.040	0.087	0.352	0.229			-0.240	0.027
15	1.395	0.561	0.019	0.213	-0.376	-0.184				
16	1.019	0.135	0.060	0.072						
17	1.067	0.742	0.147	0.069						
18	0.775	0.269	0.047	0.080						
19	0.889	0.006	0.103	0.203						
20	0.684	0.522	0.241	0.188						
21	0.891	0.754	0.229	0.267						
22	1.148	0.333	0.187	0.239						

23	0.802	-0.155	0.229	0.141						
24	1.165	0.435	0.219	0.171						
25	1.104	-0.003	0.241	0.188						
26	0.614	-0.309	0.515	0.025						
27	0.236	0.048	0.384	0.496						
28	0.264	-0.075	0.619	0.375						
29	0.421	0.039								
