# Supplementary material for: A single N-terminal amino acid determines the distinct roles of histones H3 and H3.3 in the Drosophila male germline stem cell lineage
Source: PLoS Biol. 2023 May 1;21(5):e3002098. doi: 10.1371/journal.pbio.3002098 (PMC10174566; doi:10.1371/journal.pbio.3002098)
Supplement: S4 Table — (PDF) [file pbio.3002098.s012.pdf]

S4 Table:

	H3	H3A31S	H3.3	H3.3S31A
1	0.505003	0.6234234	0.7142018	0.5588644
2	0.4652818	0.6324234	0.7142018	0.5682859
3	0.505003	0.456535	0.6414944	0.7142018
4	0.3078724	0.4123425	0.6367248	0.5420177
5	0.2699318	0.4346435	0.6737306	0.4756581
6	0.6457511	0.5567548	0.6603306	0.5732267
7	0.205099	0.5634624	0.8052035	0.623414
8	0.6373971	0.5654345	0.8331215	0.823421
9	0.5792014	0.5653452	0.8393985	0.5383276
10	0.5580345	0.6657467	0.8393985	0.5836723
11	0.676327	0.6342342	0.934834	0.625632
12	0.4770097	0.4234242	0.8623843	0.512134
13	0.3923489	0.4232433	0.962432	0.489232
14	0.645301	0.4435346	0.532423	0.7232244
15	0.6277997	0.5324234	0.6352837	0.528932
16	0.4751919	0.5234234	0.4576943	0.4857843
17	0.4662648	0.5342342	0.632983	0.6389328
18	0.3042862	0.5342342	0.7094834	0.5484387
19	0.5895384	0.6	0.8238238	0.6498438
20	0.6673401	0.6324234	0.539242	0.6398894
21	0.3457425	0.6345234	0.683822	0.7478438
22	0.393957	0.3123123	0.7948923	0.8478438
23	0.4056038	0.3435346	0.832378	0.6487433
24	0.4249862	0.5435345	0.9327833	0.5093289
25	0.4323314	0.5342343	0.8367124	0.612332

26	0.4937092	0.5324234	0.7276237	0.7326723
27	0.4723083	0.7895	0.439823	0.5378213
28	0.5664577	0.7351432	0.5309289	0.6348943
29	0.6446252	0.7513251	0.6398478	0.5328932
30	0.5344189	0.7514362	0.7478439	0.598478
31	0.5210149	0.735431		
32	0.5236257	0.6231351		
33	0.3454889	0.6587441		
34	0.4435689	0.6541122		
35	0.514042			
36	0.2873534			
37	0.3698455			
38	0.5764542			
39	0.3808914			
40	0.6064319			
41	0.5845584			
42	0.6333473			
43	0.5747869			
44	0.6112202			
45	0.1298327			
46	0.4126256			
47	0.2777169			
48	0.3379395			
49	0.4767095			
50	0.3938842			
51	0.5632984			
52	0.4171914			
