# Supplementary material for: A single N-terminal amino acid determines the distinct roles of histones H3 and H3.3 in the Drosophila male germline stem cell lineage
Source: PLoS Biol. 2023 May 1;21(5):e3002098. doi: 10.1371/journal.pbio.3002098 (PMC10174566; doi:10.1371/journal.pbio.3002098)
Supplement: S5 Table — (PDF) [file pbio.3002098.s013.pdf]

**S5 Table:**

| | H3K27me3 | | H4K20me3 | |
|---|---|---|---|---|
| | **H3 WT** | **H3A31S** | **H3 WT** | **H3A31S** |
| 1 | 1.78231818 | 1.003287 | 1.5698695 | 2.82295283 |
| 2 | 2.30611039 | 1.0038244 | 2.8875211 | 1.09981996 |
| 3 | 1.04781295 | 1.0038737 | 1.45446453 | 1.88535789 |
| 4 | 4.54749593 | 1.008937 | 2.96744882 | 1.20878192 |
| 5 | 1.51152774 | 1.0098271 | 4.22501681 | 2.15315126 |
| 6 | 2.3629232 | 1.013489 | 1.00953079 | 1.03679741 |
| 7 | 4.40181046 | 1.0167346 | 1.5560023 | 1.73120322 |
| 8 | 2.84736067 | 1.018232 | 1.92253776 | 1.03956375 |
| 9 | 2.98564541 | 1.018328 | 1.1131251 | 1.14419054 |
| 10 | 1.62566522 | 1.01893 | 3.40767437 | 1.58339691 |
| 11 | 1.64775363 | 1.0189348 | 1.80748225 | 1.29192066 |
| 12 | 1.3577281 | 1.0189823 | 1.30595238 | 1.03713 |
| 13 | 1.03939127 | 1.0198347 | 1.77630778 | 1.55950026 |
| 14 | 1.89772195 | 1.0268947 | 1.37608868 | 1.30224882 |
| 15 | 1.54647574 | 1.0283783 | 1.38984057 | 1.32723822 |
| 16 | 1.82828518 | 1.0284378 | 1.15512891 | 1.03686739 |
| 17 | 1.37648763 | 1.0284378 | 1.93292301 | 1.16063649 |
| 18 | 3.63357807 | 1.02874 | | 2.12998551 |
| 19 | 2.06851693 | 1.0378347 | | 1.06175943 |
| 20 | 1.08682106 | 1.03823 | | 1.1322978 |
| 21 | 1.88809093 | 1.047372 | | |
| 22 | 4.87522781 | 1.048923 | | |
| 23 | 1.19049149 | 1.0489238 | | |
| 24 | 7.22520586 | 1.0928732 | | |
| 25 | 1.32017942 | 1.123453 | | |
| 26 | 4.58812042 | 1.1732984 | | |
| 27 | 2.68466793 | 1.1938478 | | |
| 28 | | 1.272763 | | |
| 29 | | 1.328948 | | |
| 30 | | 1.3828387 | | |
| 31 | | 1.421878 | | |
| 32 | | 1.4621872 | | |
| 33 | | 1.8273467 | | |
| 34 | | 2.000726 | | |
| 35 | | 2.00383 | | |

| | | | | |
|---|---|---|---|---|
| 36 | | 2.0128348 | | |
| 37 | | 2.0354452 | | |
| 38 | | 2.234231 | | |
| 39 | | 2.397823 | | |
| 40 | | 2.589237 | | |
| 41 | | 2.6238236 | | |
| 42 | | 2.888937 | | |
| 43 | | 2.9237832 | | |
| 44 | | 3.0421893 | | |
| 45 | | 3.1783287 | | |
| 46 | | 3.4988987 | | |
| 47 | | 4.8214528 | | |
| 48 | | 4.924126 | | |
| 49 | | 4.993727 | | |
| 50 | | 5.213347 | | |
