# Supplementary material for: A single N-terminal amino acid determines the distinct roles of histones H3 and H3.3 in the Drosophila male germline stem cell lineage
Source: PLoS Biol. 2023 May 1;21(5):e3002098. doi: 10.1371/journal.pbio.3002098 (PMC10174566; doi:10.1371/journal.pbio.3002098)
Supplement: S6 Table — (PDF) [file pbio.3002098.s014.pdf]

S6 Table:

	H3.3 WT old signal			H3.3S31A old signal		
1	**12 hr**	**24 hr**	**36 hr**	**12 hr**	**24 hr**	**36 hr**
2	47.73618	24.84284	0.945114	14.48159	1.671011	0.720246
3	58.83411	17.23737	-0.01178	34.78527	1.282397	0.535595
4	38.23234	21.8951	0.247898	5.735546	0.547177	0.614484
5	34.02596	24.98505	2.550097	13.7881	0.990151	0.480484
6	26.62867	31.4063	2.674008	20.23622	1.501602	0.40571
7	38.89192	35.60371	2.187838	15.72423	0.401045	0.66013
8	38.04328	23.92922	5.36276	7.634003	0.334584	0.750244
9	49.57918	30.70699	5.924892	6.645663	0.474563	0.653651
10	47.1264	21.54344	3.825965	17.4165	0.138986	0.35898
11	42.67001	37.18892	2.318617	11.3745	3.922642	0.70151
12	48.234156	30.35239	0.117078	17.33183	0.249106	0.676636
13	39.285729	18.4424	-0.01056	11.4594	0.442394	0.658495
14	42.493486	20.92496	-0.34764	17.9079	0.246283	
15			2.14072	16.26656	0.256792	
16			1.452075		0.502443	
17			0.780869			
18			0.756709			
			1.237075			
