# Supplementary material for: A single N-terminal amino acid determines the distinct roles of histones H3 and H3.3 in the Drosophila male germline stem cell lineage
Source: PLoS Biol. 2023 May 1;21(5):e3002098. doi: 10.1371/journal.pbio.3002098 (PMC10174566; doi:10.1371/journal.pbio.3002098)
Supplement: S7 Table — (PDF) [file pbio.3002098.s015.pdf]

S7 Table:

	**H3**	**H3A31S**	**H3.3**	**H3.3S31A**
Promoter and TSS	0.470	0.000	0.805	0.969
Transcription elongation	0.683	0.999	0.836	0.935
Regulatory regions (enhancers)	0.930	0.780	0.953	0.612
Active introns	0.359	0.949	0.387	0.345
Active gene on the Male X	0.367	0.219	0.589	0.042
Polycomb-mediated repression	0.776	0.950	0.006	0.184
Pericentromeric heterochromatin	0.000	0.236	0.000	0.012
Heterochromatin-like embedded in euchromatin	0.094	0.930	0.101	0.928
Transcriptionally silent, intergenic	0.774	1.000	0.687	0.031
