# Supplementary material for: A single N-terminal amino acid determines the distinct roles of histones H3 and H3.3 in the Drosophila male germline stem cell lineage
Source: PLoS Biol. 2023 May 1;21(5):e3002098. doi: 10.1371/journal.pbio.3002098 (PMC10174566; doi:10.1371/journal.pbio.3002098)
Supplement: S8 Table — (PDF) [file pbio.3002098.s016.pdf]

S8 Table:

	WT H3 SG	WT H3 GSC
1	0.738	0.505003
2	0.673467	0.465282
3	0.484	0.505003
4	0.84743	0.307872
5	0.732782	0.269932
6	0.5636	0.645751
7	0.63256	0.205099
8	0.784578	0.637397
9	0.7256	0.579201
10	0.6256	0.558035
11	0.897	0.676327
12	0.478578	0.47701
13	0.57223	0.392349
14	0.58734	0.645301
15	0.73273	0.6278
16	0.63287	0.475192
17	0.73287	0.466265
18	0.7389	0.304286
19	0.43287	0.589538
20	0.8287	0.66734
21		0.345743
22		0.393957
23		0.405604
24		0.424986
25		0.432331
26		0.493709
27		0.472308
28		0.566458
29		0.644625
30		0.534419
31		0.521015
32		0.523626
33		0.345489
34		0.443569
35		0.514042
36		0.287353

37		0.369846
38		0.576454
39		0.380891
40		0.606432
41		0.584558
42		0.633347
43		0.574787
44		0.61122
45		0.129833
46		0.412626
47		0.277717
48		0.33794
49		0.47671
50		0.393884
51		0.563298
52		0.417191
