# Supplementary material for: A single N-terminal amino acid determines the distinct roles of histones H3 and H3.3 in the Drosophila male germline stem cell lineage
Source: PLoS Biol. 2023 May 1;21(5):e3002098. doi: 10.1371/journal.pbio.3002098 (PMC10174566; doi:10.1371/journal.pbio.3002098)
Supplement: S9 Table — (PDF) [file pbio.3002098.s017.pdf]

S9 Table:

	H3 WT	H3A31S
1	-0.4478596	0.406708726
2	-0.924558112	1.117425832
3	-1.198680742	-0.723181046
4	0.990116984	-0.50036942
5	0.655686219	0.031450425
6	-1.986923903	-0.602375231
7	0.353342526	-0.193902611
8	0.4027792	-0.299776201
9	-0.325793034	-0.045783389
10	0.010714577	-0.21484731
11	0.111028617	0.183169015
12	-0.166668662	-0.268065661
13	-0.058604978	-0.035421169
14	0.416207966	-0.661338624
15	0.227022941	-0.496603782
16	-0.731326001	-0.760416624
17	0.357600768	-0.325258945
18		0.076361316
19		-0.045195113
20		0.026605742
