# Supplementary material for: A single N-terminal amino acid determines the distinct roles of histones H3 and H3.3 in the Drosophila male germline stem cell lineage
Source: PLoS Biol. 2023 May 1;21(5):e3002098. doi: 10.1371/journal.pbio.3002098 (PMC10174566; doi:10.1371/journal.pbio.3002098)
Supplement: S10 Table — The 3D quantification of old histone [sum intensity (a.u)] was performed in GSCs at each time point (i.e., at 20 hours and 30 hours) after heat shock. Individual data points represent individual GSCs at each time point. GSC, germline stem cell. (PDF) [file pbio.3002098.s018.pdf]

**S10 Table:**

| | WT H3.3 | | H3.3S31S | |
|---|---|---|---|---|
| | **20 hrs** | **30 hrs** | **20 hrs** | **30 hrs** |
| 1 | 1226010 | 927737 | 1184170 | 1064420 |
| 2 | 1334770 | 865927 | 1942650 | 936547 |
| 3 | 1411710 | 818985 | 1402930 | 725623 |
| 4 | 995265 | 863537 | 2030000 | 677600 |
| 5 | 1098360 | 823895 | 1370000 | 418604 |
| 6 | 1306620 | 870571 | 1760000 | 548152 |
| 7 | 1201800 | 580663 | 1930000 | 849218 |
| 8 | 1368060 | 919741 | 1253610 | 1214720 |
| 9 | 1313300 | 1028840 | 1390000 | 1184170 |
| 10 | 991347 | 696327 | 2026820 | 1106000 |
| 11 | 941379 | 716940 | | |
