# Supplementary material for: A single N-terminal amino acid determines the distinct roles of histones H3 and H3.3 in the Drosophila male germline stem cell lineage
Source: PLoS Biol. 2023 May 1;21(5):e3002098. doi: 10.1371/journal.pbio.3002098 (PMC10174566; doi:10.1371/journal.pbio.3002098)
Supplement: S1 Raw Images — (PDF) [file pbio.3002098.s024.pdf]

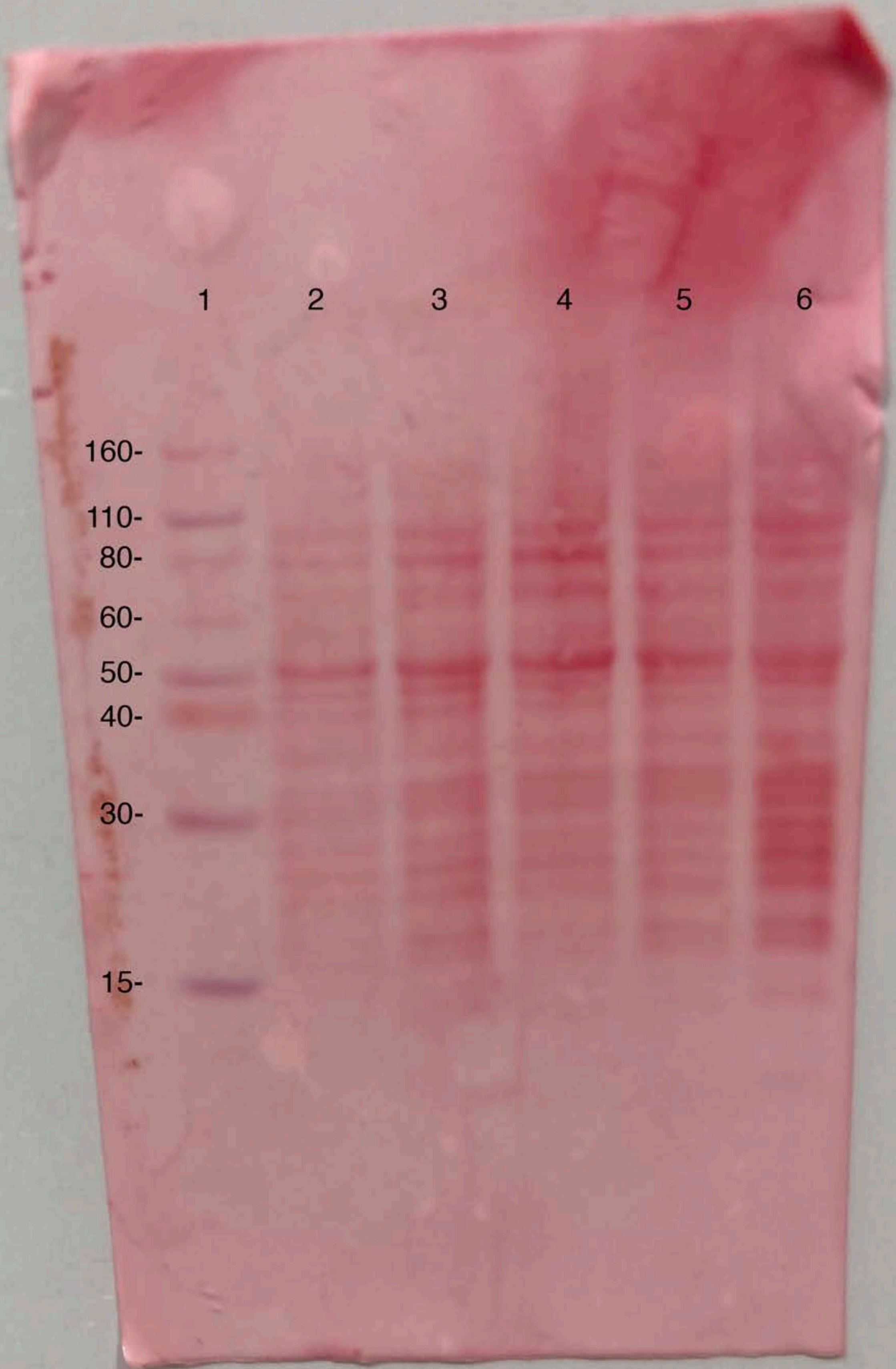

1: Ladder
2: Nos > Upd (control)
3: Nos > Upd, H3-eGFP
4: Nos > Upd, H3A31S-eGFP
5: Nos > Upd, H3.3-eGFP
6: Nos > Upd, H3.3S31A-eGFP

Ponceau staining of blot used for H3 and GFP western blots
Corresponding to Figure S7, panel 1
All lanes loaded with 5 pairs of Nanos-driven Unpaired (Upd) testis tumors
Image acquired using iPhone

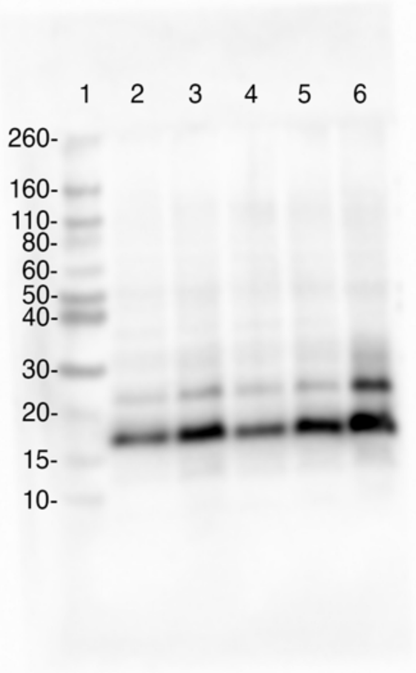

1: Ladder

2: Nos > Upd (control)

3: Nos > Upd, H3-eGFP

4: Nos > Upd, H3A31S-eGFP

5: Nos > Upd, H3.3-eGFP

6: Nos > Upd, H3.3S31A-eGFP

anti-Histone H3 western blot
Corresponding to Figure S7, panel 2
All lanes loaded with 5 pairs of Nanos-driven Unpaired (Upd) testis tumors
Image acquired using Chemi rapid mode on Syngene G:Box XRQ

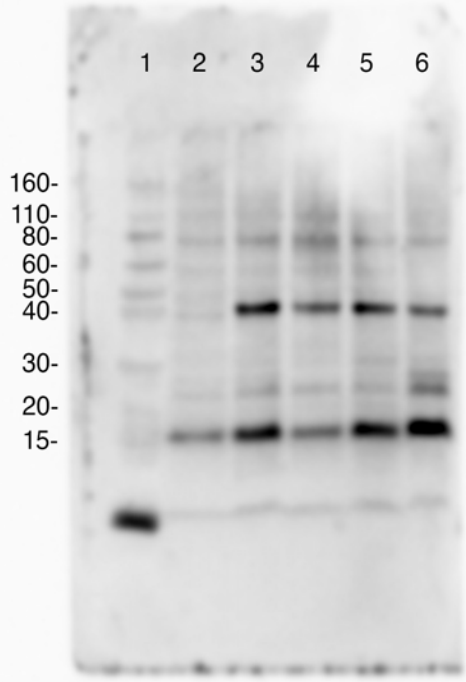

1: Ladder
2: Nos > Upd (control)
3: Nos > Upd, H3-eGFP
4: Nos > Upd, H3A31S-eGFP
5: Nos > Upd, H3.3-eGFP
6: Nos > Upd, H3.3S31A-eGFP

anti-GFP western blot
Corresponding to Figure S7, panel 3
All lanes loaded with 5 pairs of Nanos-driven Unpaired (Upd) testis tumors
Image acquired using Chemi rapid mode on Syngene G:Box XRQ

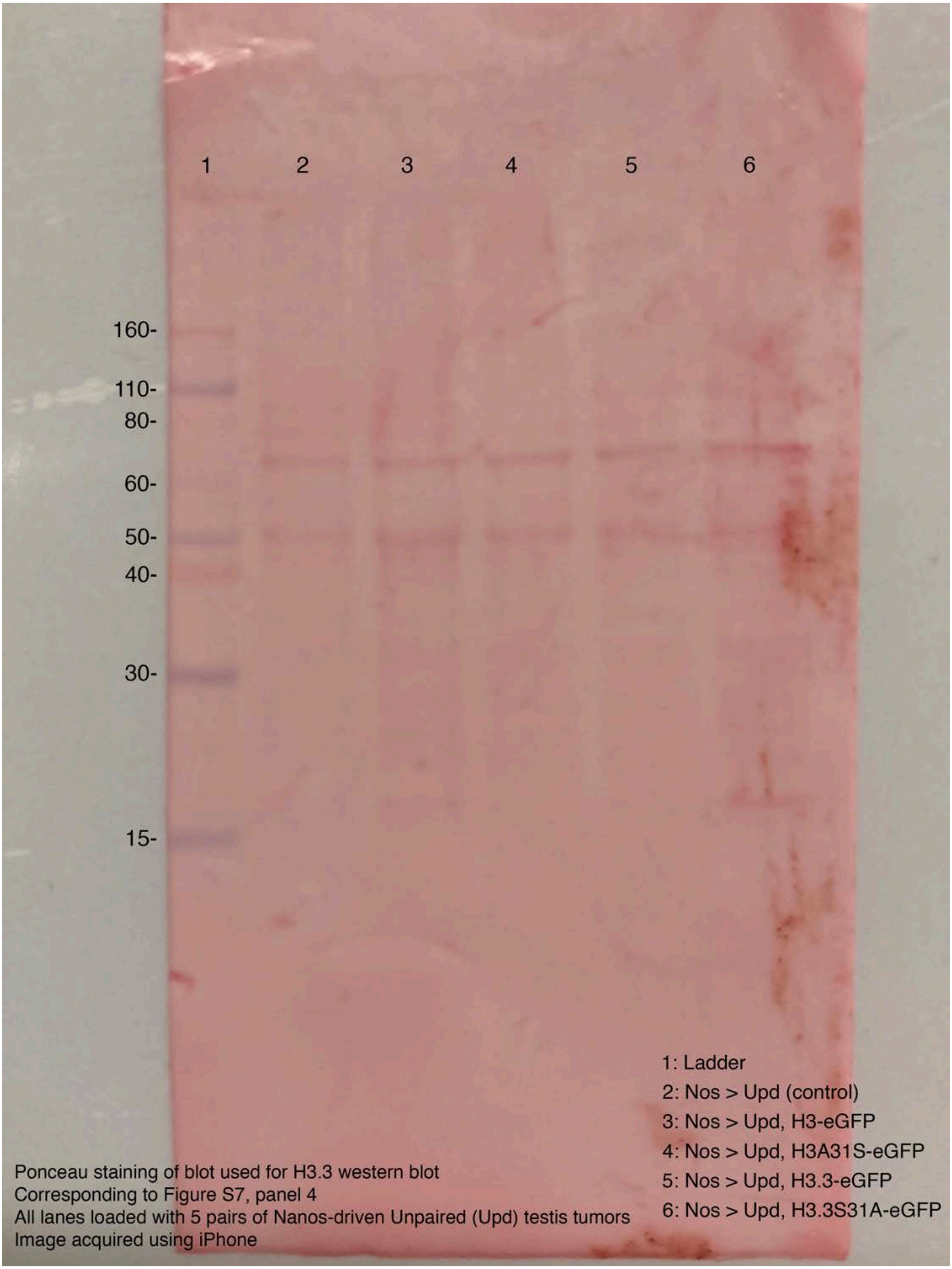

1
2
3
4
5
6
160-
110-
80-
60-
50-
40-
30-
15-
1: Ladder
2: Nos > Upd (control)
3: Nos > Upd, H3-eGFP
4: Nos > Upd, H3A31S-eGFP
5: Nos > Upd, H3.3-eGFP
6: Nos > Upd, H3.3S31A-eGFP
Ponceau staining of blot used for H3.3 western blot
Corresponding to Figure S7, panel 4
All lanes loaded with 5 pairs of Nanos-driven Unpaired (Upd) testis tumors
Image acquired using iPhone

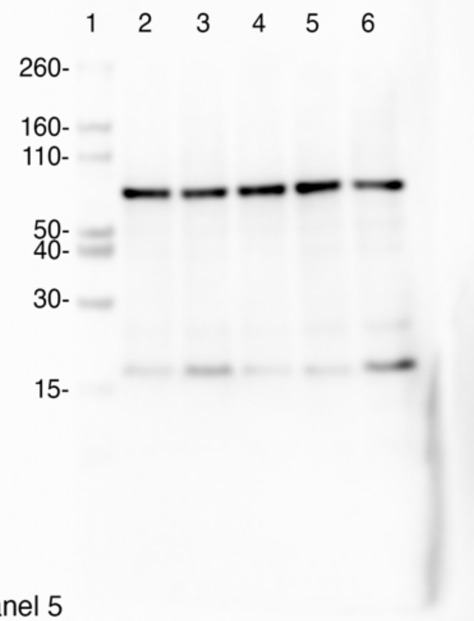

1: Ladder
2: Nos > Upd (control)
3: Nos > Upd, H3-eGFP
4: Nos > Upd, H3A31S-eGFP
5: Nos > Upd, H3.3-eGFP
6: Nos > Upd, H3.3S31A-eGFP

anti-Histone H3.3 western blot
Corresponding to Figure S7, panel 5
All lanes loaded with 5 pairs of Nanos-driven Unpaired (Upd) testis tumors
Image acquired using Chemi rapid mode on Syngene G:Box XRQ
