# Supplementary material for: A single N-terminal amino acid determines the distinct roles of histones H3 and H3.3 in the Drosophila male germline stem cell lineage
Source: PLoS Biol. 2023 May 1;21(5):e3002098. doi: 10.1371/journal.pbio.3002098 (PMC10174566; doi:10.1371/journal.pbio.3002098)
Supplement: Attachment — Submitted filename: Responses to Reviewers.pdf [file pbio.3002098.s025.pdf]

Reviewer #1: This study uses the powerful Drosophila male germline stem cell (GSC) system to probe the function of the 31 residue of canonical replicative histone H3 and the histone H3 variant H3.3. Intriguingly, this is the only residue that differs between the N terminus of H3 and H3.3. The authors strategy is to (over) express GFP-tagged versions of wild type H3 or mutant H3A31S, and wild type H3.3 or H3.3S31A in GSCs and early germ cells. They subsequently monitor effects on GSC maintenance, testes development and fertility. In addition, using their previously published genetic system that allows pre-existing and newly synthesised versions of proteins to be distinguished, the authors track the fate of new and old wild type or mutant histones during asymmetry cell division (ACD). The goal is to determine whether residue 31 functions in the asymmetric distribution of histones that has been shown to occur in GSC and daughter (gonialblast; GB) cells. This study is novel and interesting in that it addresses the function of amino acid 31 of H3/H3.3 in the context of a developing tissue. The genetic switch assays utilised, combined with live imaging in testes, are sophisticated and cutting edge. I would recommend this report for publication after addressing the following 3 major points:

We thank this reviewer for the overall very positive comments!

Here we are addressing all three major points in the following responses.

1. One general query I have relates to the expression level of wild type H3 or mutant H3A31S, and wild type H3.3 or H3.3S31A in the different experiments. For example, was the expression level comparable between H3 and H3A31S? The authors should at least provide some estimation of the level of GFP-tagged wild type/mutant histone relative to endogenous H3 expression. Also in Figure 7 while the incorporation pattern of the tagged, over-expressed versions of H3/H3.3 are considered, were any changes to endogenous H3 pattern noted upon transgene expression?

We thank this reviewer for a great question. Previously, we analyzed the H3-GFP transgene expression in the same genetic background used in Figure 7 (*nos-Gal4>UAS-H3-GFP, UAS-upd*), in Figure S7 of a previous publication [1]. There, the relative levels of endogenous H3 and transgenic H3-GFP were accessed using both anti-GFP and anti-H3 antibodies. However, with the immunoblot experiments, it is not possible to compare the levels of the exogenous H3-GFP (driven by the germline-specific *nos-Gal4*) with the endogenous H3 (from all cells in the testis sample) as they have different cell type specificities. Only a subset of cells (i.e., germ cells) express the histone-GFP transgene in the testis samples. Based on these results, the transgene does not seem to affect endogenous H3 levels [compare the endogenous H3 bands in *nos-Gal4>UAS-H3-GFP, UAS-upd* (*nos>upd, H3*) vs. the control *nos-Gal4>UAS-upd* (*nos>upd*)].

To address this point in the current manuscript, we performed a similar immunoblot experiment to query the expression levels of the endogenous histones in different transgenic lines, as well as the expression levels of the different transgenes (a new Figure S7). When blotting for histone H3, we observe no obvious differences in levels of endogenous H3 when comparing wild-type with mutant H3A31S transgenic lines. Similarly, when blotting for H3.3, we detected comparable levels of endogenous H3.3 when comparing wild-type with mutant H3.3S31A transgenic lines. Additionally, levels of endogenous histone do not seem to be affected by the transgenes when comparing genotypes with transgene (i.e., *nos>upd*, *H3* or *H3.3* or *H3A31S* or *H3.3S31A*) *versus* the genotype without any histone transgene (*nos>upd*). When blotting for GFP, exogenous H3/H3A31S-GFP was detected at a similar level between wild-type H3 and mutant H3A31S transgenic lines, as well as between wild-type H3.3 and mutant H3.3S31A transgenic lines.

We agree with the reviewer that it would be informative to know the incorporation patterns of the transgenic histones in comparison to the endogenous histones. As stated above, the transgenic H3 and H3.3 are expressed exclusively in the germ cells while endogenous H3 and H3.3 are present in all cell types including germ cells and somatic cells. Therefore, while it is straightforward to profile cell-specific incorporation patterns of the transgenic histones by targeting GFP, there is currently no method to distinguish endogenous H3 or H3.3 from that of the transgenic H3 or H3.3 in a comparable cell-specific manner. Thus, by profiling endogenous histone by any type of chromatin profiling method, such as ChIC-seq, ChIP-seq, or CUT&RUN, not only would we lose cell-specific information on incorporation patterns, but also the transgene that provides cell-specificity prevents us from distinguishing between endogenous and transgenic histones.

2. In Figure 3, the authors show that old H3A31S displays less asymmetry compared to wild type old H3, concluding that Ala31 is required for asymmetry. This observation is further supported by data presented in Figure 5 in which parental H3K27me3 redistribution is assessed. According to the quantitation, the H3K27me3 distribution pattern is disrupted. However, it is perhaps a leap to make the general conclusion that at the replication fork is disrupted, at least without validation by an independent method. For example, it is possible that the pattern is disrupted as H3K27 methylation status is affected by the H3A31S mutation or its over expression. For this reason the claim the old histone recycling is disrupted should be toned down. Also in this Figure, can the authors explain why EdU coats only one stand in 5A, but both strands in 5B? Finally, PCNA staining should be included in 5B.

We thank this reviewer for the very insightful question and suggestions. We have toned down the conclusion for Figure 5 to be on the specific histone modifications enriched on old histones. In the revision, we used an alternative histone modification enriched with old histone H4 (anti-H4K20me3) [2-4] on chromatin fibers derived from *nos>H3A31S-GFP* testes, along with EdU and anti-PCNA (Figure 5C-D, 5F).

Additionally, our previous and ongoing studies indicate that the chromatin fibers derived from early-stage germ cells, such as the *nos>H3-FP* labeled ones, often display delayed lagging strand synthesis and/or processing. This is reflected by the longer time to accomplish the lagging strand, manifested by asymmetric EdU incorporation, as well as asymmetric distribution of the lagging strand-enriched PCNA and lagging strand-specific RPA-70, a highly conserved single-stranded DNA-binding protein [5]. In contrast, such asymmetries of EdU and PCNA were much less observed in symmetrically dividing cultured Kc cells [6]. Interestingly, these asymmetries become less obvious on the chromatin fibers derived from H3A31S-expressing early-stage germ cells (Figure 5B, 5D). In these new experiments, we did stain the H3A31S-labeled fibers with PCNA and observed less asymmetric PCNA distribution (Figure 5D) compared to WT H3-labeled fibers (Figure 5C and 5A). We also added a Figure S4, where we show distribution of EdU pulse labeling on the replicative chromatin fibers derived from early-stage germ cells expressing WT H3 vs. mutant H3A31S. These data indicate that the EdU asymmetry is less on H3A31S-labeled chromatin fibers than on WT H3-labeled chromatin fibers.

3. In Figure 3, the authors show that old H3.3S31A is symmetrically distributed, comparable to wild type H3.3, concluding Ser31 is not critical for asymmetry. This observation is further supported by the claim in Figure 6 that H3.3S31A turnover is faster than that of wild type H3.3.

However, an alternative explanation is that H3.3S31A is more sensitive to fixation. Live snapshot should be quantified to exclude this possibility.

We have performed live snapshots for old H3.3S31A vs. old H3.3 after heat shock-induced tag switch and plotted their turnover using live cell image (Figure S5), which show consistent results as using fixed samples.

Other minor points:

In Figure 1, the authors convincingly show that compared to expression of H3, H3A31S expression in testes leads to an over-population of early germ cells (including GSCs). What is not clear is whether over-expression of H3 alone can drive this effect i.e. what is the basal number of GSCs in the control line? It is also not clear why only 33% of testes analysed showed this phenotype (Fig 1E). The authors should provide an explanation.

Many previous publications showed that male GSC number/testis from lab wild-type strains is on average of ~ 8-10 per testis [7-10], similar to the number (8.6 ± 0.3) we obtained in H3-expressing testes (n=30, Figure 1D).

Additionally, we only classified testes with obviously expanded progenitor germ cell zone as the ones with early germline tumor phenotype (Figure 1D). Therefore, the 33% quantification in Figure 1F reflects the penetrance of this phenotype, since testes with less developed tumor were not counted. Notably, penetrance applies for almost all genetics experiments as it is very rare that a certain phenotype reaches 100% penetrance. We added more explanation to these results.

In Figure 2, the authors convincingly show that compared to expression of H3.3, H3.3S31A expression in testes leads to a gradual loss of GSCs that correlate well with a decline in fertility over time. Notably, even in the line expressing H3.3 wild type, GSCs were reduced after 10 days. My guess is that this is due to ageing, but the authors should address this point. Figure 2D and 2E show a reduced level of Stat92E present in H3.3S31A GSCs. For the quantitation in 2E, it appears that some GSCs showed no Stat92E signal. Perhaps the authors can expand on whether a reduction in Stat92E signal is sufficient to alter GSC identity or whether a total loss is required?

This reviewer is absolutely right regarding the age-dependent decrease of male fertility. We now added more clarification on this point: “Age-dependent decrease of GSC activity and male fertility has been reported previously [1, 11-14].”

The variation of the Stat92E signals should be due to technical issues using the immunostaining with this anti-Stat92E antibody, as we observed previously [15]. In these experiments, immunostaining with anti-Stat92E was performed side-by-side on both genotypes. All data acquisition and quantification were done in parallel in an unbiased manner for all GSCs. And the same criterion for data analyses applies to both genotypes.

In Figure 4E, the calculated Spearman's rank correlation for H3A31S and H3.3S31A appear similar. Yet from the images presented in 4E, the degree of overlap does not appear that similar. Are the values for H3A31S and H3.3S31A significantly different?

We have now shown more representative images in this figure. We have also added statistical analyses to compare the Spearman's rank correlation between the two mutant groups, which showed no significant statistical difference.

Typo Figure 7A 'targeting'

We have changed it in the figure panel.

Typo Figure 1 legend B 'in testes' Corrected.

Reviewer #2: In this work, Chandrasekhara et al., use the Drosophila male germline stem cell system to study the molecular mechanism determining the distribution pattern of new vs old histones. Taking the advantage of different incorporation patterns between histone H3 and H3.3 (with the former being asymmetric and the latter randomly incorporating between two sister chromatids), they identified the difference of the 31st amino acid (A in H3 and S in H3.3) is critical for determining different segregation patterns towards two sister chromatids in germ cells. By swapping the 31st amino between these histones, they found that H3's 31st A residue is critical for its preferential incorporation toward one of the sisters during S phase, whereas H3.3's 31st S is critical for regulating turnover time of H3.3 to continuously replace preexisting histones. Moreover, they show that one of the replicating strands which is likely incorporating old histones contains more H3K27me3 marks.

Furthermore, they conducted ChIC seq to show where these ectopically expressed histones H3, H3.3 and their swapped versions bind in the genome.

These findings are certainly of interest to the broad readership of Plos Biology. However, some results presented here are very difficult to interpret, especially the ChIC seq. I would suggest to clarify these uncertain points for better understanding of what happening in the cells.

We thank this reviewer for the overall positive comments! Here we are addressing each of the main and minor points in the following responses.

Main points;

1) The authors show that H3.3S31A and H3A31S both show phenotypes possibly due to altered incorporation patterns of new/old molecules toward two sister chromatids. Do these swapped versions still keep same replication dependency/independency?

This is a great question. We examined the time required for genome-wide incorporation of new histones: If the new histone incorporation is continuous, then the incorporation mode is replication-independent; if the new histone incorporation only becomes detectable after a full-term of S phase, then the incorporation mode is most likely replication-dependent. Based on these assays, we found that the incorporation of new H3A31S is still replication-dependent, just like wild-type H3; and the incorporation of new H3.3S31A is still replication-independent, just like wild-type H3.3. We now added these additional points to the Results (Figure S1).

2) Based on a previous report from same group (https://nam02.safelinks.protection.outlook.com/?url=https%3A%2F%2Fwww.ncbi.nlm.nih.gov%2Fpmc%2Farticles%2FPMC6684448%2F&data=05%7C01%7Cxchen32%40jhu.edu%7Cfcd66d08c82a4191a34d08dab1173f70%7C9fa4f438b1e6473b803f86f8aedf0dec%7C0%7C0%7C638017010664372377%7CUnknown%7CTWFpbGZsb3d8eyJWIjoiMC4wLjAwMDAiLCJQIjoiV2luMzIiLCJBTiI6Ik1haWwiLCJXVCI6Mn0%3D%7C3000%7C%7C%7C&sdata=EM6brwWDWSiyUHHn1Etn6p3NVovLqQ8P2%2FyO%2FIsgpa0%3D&reserved=0), asymmetric incorporation of H3 globally to the entire length of a sister chromosome depends on the unidirectional replication mechanism. Does H3A31S disrupt unidirectional replication or do the authors think another mechanism is at play?

This is another great question. First, we want to clarify that the histone asymmetry is not an all-or-nothing phenomenon, even though the asymmetry is apparent and significant, as shown in Figure 3 [e.g., Old H3, GSC 1.91 ± 0.06 (n = 29)]. However, we are not sure whether this global

H3 asymmetry has any chromosomal specificity or applies to the entire length of a chromosome. Second, as this reviewer pointed out, we previously found increased incidence of unidirectional replication fork movement on chromatin fibers derived from early-stage germ cells and DNA fibers derived from early-stage germ cell-enriched testis samples [5]. However, currently the chromatin fiber data do not have germline stage specificity between the germline stem cells and the early germline progenitor cells, due to the early germline driver *nanos-Gal4* used to drive the histone-FP transgene to label and recognize the chromatin fibers. In addition, the DNA fibers are lack of either cell type (i.e., germline *versus* somatic gonadal cells) or germline stage (i.e., stem cells *versus* early progenitor cells) specificities, since they are depleted of any DNA associated proteins during the DNA fiber preparation procedures. We are currently working rigorously to improve the precision to examine replication fork movement on DNA fibers specifically derived from germline stem cells. However, this is beyond the scope of this work.

As to this work, we have shown that at individual replication fork, the asymmetric old histone re-incorporation has been compromised in the H3A31S-expressing early germ cells, using the old H3-enriched H3K27me3 and old H4-enriched H4K20me3 (Figure 5). We hypothesize that asymmetric old histone recycling is the key to establish asymmetric histone patterns in S phase, since new H3 fill in “gaps” after old histones take their places [5, 16]. If this very first step of histone asymmetry is disrupted by expression of the mutant H3A31S, there should be no asymmetry between sister chromatids even with coordinated replication fork movement.

In summary, this great question and suggestion will be studied in the future with more details.

3) In the Fig4 colocalization assay, do the authors consider H3 and H3.3 visualized here are all on DNA? Do these images show any fraction in the nucleoplasm? Colocalization with DNA may help to interpret the data.

In all these experiments, we used detergent such as Triton X-100 (0.1%) for multiple washes after tissue fixation (see Materials and Methods), with which most of the free histones should be washed off from the sample, as shown previously [5, 15, 17, 18].

In addition, we now included imaging results of old and new histones with a DNA dye in a new supplemental figure (Figure S2), where both populations of histones are positive with DNA dye.

4) Same figure (Fig4). Do the authors think the distinct localization of old/new H3 occurs in any germ cell or only GSCs?

We now included symmetrically dividing spermatogonial cells (SGs), which display higher degree of overlapping between old and new H3 than that of GSCs, indicating that the distinct localization of old vs. new H3 occurs specifically in GSCs (Figure S3).

5) Same figure again (Fig4). Do the authors still see a distinct localization pattern in H3T3A? If it was reported previously, please describe.

This is a very insightful question. Indeed, in our previous report, we have shown that expression of a mutant histone H3T3A disrupts asymmetric old and new H3 inheritance [Figure 4, [1]], but does not affect the differential distribution of old vs. new H3 in GSCs at prophase [Figure 3N, [1]]. This is because H3T3A acts dominant negatively to abolish the phosphorylation of the Thr3 residue of histone H3, which is a histone modification specifically detectable in the mitotic GSCs. Therefore, this mutant histone disrupts the asymmetric recognition and segregation of

sister chromatids enriched with old *versus* new H3 during mitosis, but not the establishment of the histone asymmetry established during S phase, which results in randomized histone inheritance in anaphase and telophase GSCs but not the differential condensation in prophase and prometaphase GSCs [19].

6) Why does Figure5 show Edu in a single strand on the top and both strands on the bottom? Does this mean the DNA synthesis pattern is different between them? A couple of typos here; PCNA, H3 WT in B'.

We thank this reviewer for this insightful question. Indeed, our previous and ongoing studies indicate that the chromatin fibers derived from early-stage germ cells, such as the *nos>H3-FP* labeled ones, often display delayed lagging strand synthesis and/or processing. This is reflected by the longer time to accomplish the lagging strand, manifested by asymmetric EdU incorporation, as well as asymmetric distribution of the lagging strand-enriched PCNA and lagging strand-specific RPA-70, a highly conserved single-stranded DNA-binding protein [5]. In contrast, such asymmetries of EdU and PCNA were much less observed in symmetrically dividing cultured Kc cells [6]. Interestingly, these asymmetries become less obvious on the chromatin fibers derived from H3A31S-expressing early-stage germ cells (Figure 5B, 5D). In these new experiments, we did stain the H3A31S-labeled fibers with PCNA and observed less asymmetric PCNA distribution (Figure 5D) compared to WT H3-labeled fibers (Figure 5C and 5A). We also added a Figure S4, where we show distribution of EdU pulse labeling on the replicative chromatin fibers derived from early-stage germ cells expressing WT H3 vs. mutant H3A31S. These data indicate that the EdU asymmetry is less on H3A31S-labeled chromatin fibers than on WT H3-labeled chromatin fibers.

We have corrected those typos for Fig. 5B’ panel.

7) ChIC seq—do Upd-induced tumor cells incorporate new/old H3 differently toward two sisters? Any previous report or data is helpful.

We thank this reviewer for another very insightful question. We validated the use of the Upd-induced tumor cells as a proxy to understand histone behavior in wild-type germline stem cells (GSCs). Indeed, our ongoing studies focus on old and new histone incorporation patterns using genome-wide methods. As part of our validation, we used a similar co-localization analysis as presented in Figure 4E to measure old and new H3 in Upd overexpression-induced tumor cells at prophase to prometaphase. Our results indicate separation of old and new H3 to a degree comparable to wild-type GSCs. This is the main reason we used Upd overexpression-induced tumor cells to study the chromatin status, because the increased GSC-like cell number in this genetic background allow us to perform genomic experiments. The detailed data showing the comparable cellular features between Upd-induced tumor cells and wild-type GSCs will be for another manuscript in preparation. However, we can disclose that the data provide support for the Upd-induced tumor cells as incorporating old *versus* new H3 differently toward the two sisters in a manner that is like what is observed in wild-type GSCs. This manuscript will further report the regulation of DNA replication in old *versus* new histone deposition in the future.

8) ChIC seq—Why does H3 show distinct peaks for these regions? Is there any effect of endogenous H3? It will be helpful if H3 or H3.3's general ChIP seq pattern is compared with their data is explained.

We apologize that our description of the analysis was unclear and have included additional text and panels in Figure 7 to walk the reader through our process. We summarize our changes here. First, there is genome-wide incorporation of all tagged histones (Fig. 7B). While there are no distinct peaks between H3 and the H3A31S mutant, there is an enrichment of one over the other in some locations. We next used the ModENCODE chromatin state classifications to see if certain environments were prone to differences in histone enrichment. We looked within the nine chromatin states for differential enrichment of the wild-type histone compared with the mutant. The data from this is shown in (Figure 7C-D). To demonstrate the differences in histone enrichment, we included addition average density plots of histone occupancy within select states (Figure 7E).

We thank this reviewer for a great question. Previously, we analyzed the H3-GFP transgene expression in the same genetic background used in Figure 7 (*nos-Gal4>UAS-H3-GFP*, *UAS-upd*), in Figure S7 of a previous publication [1]. There, the relative levels of endogenous H3 and transgenic H3-GFP were accessed using both anti-GFP and anti-H3 antibodies. However, with the immunoblot experiments, it is not possible to compare the levels of the exogenous H3-GFP (driven by the germline-specific *nos-Gal4*) with the endogenous H3 (from all cells in the testis sample) as they have different cell type specificities. Only a subset of cells (i.e., germ cells) express the histone-GFP transgene in the testis samples. Based on these results, the transgene does not seem to affect endogenous H3 levels [compare the endogenous H3 bands in *nos-Gal4>UAS-H3-GFP*, *UAS-upd* (*nos>upd*, *H3*) vs. the control *nos-Gal4>UAS-upd* (*nos>upd*)].

To address this point in the current manuscript, we performed a similar immunoblot experiment to query the expression levels of the endogenous histones in different transgenic lines, as well as the expression levels of the different transgenes (a new Figure S7). When blotting for histone H3, we observe no obvious differences in levels of endogenous H3 when comparing wild-type with mutant H3A31S transgenic lines. Similarly, when blotting for H3.3, we detected comparable levels of endogenous H3.3 when comparing wild-type with mutant H3.3S31A transgenic lines. Additionally, levels of endogenous histone do not seem to be affected by the transgenes when comparing genotypes with transgene (i.e., *nos>upd*, *H3* or *H3.3* or *H3A31S* or *H3.3S31A*) *versus* the genotype without any histone transgene (*nos>upd*). When blotting for GFP, exogenous H3/H3A31S-GFP was detected at a similar level between wild-type H3 and mutant H3A31S transgenic lines, as well as between wild-type H3.3 and mutant H3.3S31A transgenic lines.

We agree with the reviewer that it would be informative to know the incorporation patterns of the transgenic histones in comparison to the endogenous histones. As stated above, the transgenic H3 and H3.3 are expressed exclusively in the germ cells while endogenous H3 and H3.3 are present in all cell types including germ cells and somatic cells. Therefore, while it is straightforward to profile cell-specific incorporation patterns of the transgenic histones by targeting GFP, there is currently no method to distinguish endogenous H3 or H3.3 from that of the transgenic H3 or H3.3 in a comparable cell-specific manner. Thus, by profiling endogenous histone by any type of chromatin profiling method, such as ChIC-seq, ChIP-seq, or CUT&RUN, not only would we lose cell-specific information on incorporation patterns, but also the transgene that provides cell-specificity prevents us from distinguishing between endogenous and transgenic histones.

Minor:

Where whole mount vs squash methods used?

All immunostaining for main figures (Figures 1, 2, 4, 6) used whole mount method. Immunostaining for Figure S6 used squash method. We now added these details to Materials and Methods in the Supplemental Material.

Reviewer #3: This manuscript reports a role for the amino acid at position 31 of histones H3 and H3.3 in inheritance of H3 during the asymmetric cell division of Drosophila male germline line stem cells (GSCs). Previous studies by this group have established that during this asymmetric cell division histones H3 is preferentially inherited by the GSC while histone H3.3 is not. This is a fascinating problem with important implications for the role of histone inheritance in acquisition of cell identity during asymmetric cell divisions. This study now identified the sequence difference between H3 and H3.3 that dictate their distinct inheritance patterns and also reports on the global localization patterns of H3 and H3.3 in GSC-like cells. The authors report the following main findings. They construct inducible H3A31S and H3.3S31A mutant histone lines and using these reagents they observe that early-stage germ cells are over-populated in the H3A31S-expressing testes while there is a loss of germ cells in testes expressing H3.3S31A. They further show that asymmetric H3 inheritance is disrupted in the H3A31S-expressing GSCs due to mis-incorporation of old histones between sister chromatids during DNA replication, H3.3S31A mutation leads to increased old histone turnover in the GSCs, and using a chromatin immune-cleavage assay show that H3A31S has enhanced occupancy at promoters of active genes while H3.3S31A is more enriched at transcriptionally silent intergenic regions compared to H3.3. From a technical point of view, the authors provide old and new H3 and H3.3 localization data based on new live cell image and 3D reconstruction that confirms their previous findings based on fixed cell imaging data. The results indicate an important new role for the N termini of histone H3 and H3.3 in the regulation of their localization and asymmetric histone inheritance and will be of great interest to the field. The manuscript is in principle suitable for publication in Plos Biology after the authors address the following minor points regarding data presentation.

We appreciate this reviewer's positive comments! Here we provide further explanations and clarifications on these important points raised by this reviewer.

1. The authors should describe in the text and Figures 1 and 2 legends how they are identifying GSCs and other cell types. The location of the niche and the hub cell indicated by asterisks in various figures and what various staining events indicate should be explained to allow a general audience to follow the results.

We now include a cartoon detailing the anatomy and different cell types at the apical tip of testis in the main figure (Figure 1A). We also added detailed description in Figure 1A legend.

2. The effects that the authors show in Figure 3 (panels A-D) look small and at least visually the asymmetric inheritance of H3 in GSCs doesn't look as impressive as what the authors have reported previously or show by quantification of live cell images in panel F. Perhaps the authors can present 3D image reconstructions (as collapsed optical stacks) of relevant GSCs in panels A to D that better show the asymmetric inheritance and its loss in H3A31S etc.

We thank this reviewer for this great suggestion. We have now revised these panels using the 3D reconstructed images. All data analyses shown in Figure 3F were performed with 3D quantification, as shown in Figure 3E and previous publications [17, 20].

3. In Figure 4, the S10P signal for mitotic chromosomes seems asymmetric in the GSCs. This seems unexpected. Can the authors explain this observation? Would it make sense to normalize the GFP and red signal to the H3S10P signal?

This reviewer is absolutely correct and raises a very intriguing question. In fact, we did show in a recent publication that H3S10P is preferentially co-localized with old H3 in GSCs from early prophase to prometaphase. By contrast, no such preferential association of H3S10P with old *versus* new H3 was detected in progenitor germ cells at the equivalent stages in mitosis [17]. Interestingly, these imaging-based results are consistent with previous biochemistry results showing that old *versus* new histones carry distinct post-translational modifications. For example, phosphorylation of many residues including Ser10 (H3S10P) is enriched on old H3 in human cells using mass spectroscopy [21]. However, this differential phosphorylation on old vs. new histones is specific to the WT H3. The two mutant histones (i.e., H3A31S and H3.3S31A) and the WT H3.3 do not display detectable separation between old and new histones, as well as any preferential co-localization of old vs. new histones with the H3S10P signals, in GSCs from early prophase to prometaphase.

4. In Figure 5, the EdU signal seems asymmetric in the chromatin fibers shown in panel A (WT H3) but is symmetrical for the fiber example shown in panel B (H3A31S testes). Can the author provide an explanation for this difference? Also, the legend should describe the reasoning for staining for PCNA (I assume as an indicator of the lagging strand), EdU, etc.

We thank this reviewer for this insightful question. Indeed, our previous and ongoing studies indicate that the chromatin fibers derived from early-stage germ cells, such as the *nos>H3-FP* labeled ones, often display delayed lagging strand synthesis and/or processing. This is reflected by the longer time to accomplish the lagging strand, manifested by asymmetric EdU incorporation, as well as asymmetric distribution of the lagging strand-enriched PCNA and lagging strand-specific RPA-70, a highly conserved single-stranded DNA-binding protein [5]. In contrast, such asymmetries of EdU and PCNA were much less observed in symmetrically dividing cultured Kc cells [6]. Interestingly, these asymmetries become less obvious on the chromatin fibers derived from H3A31S-expressing early-stage germ cells (Figure 5B, 5D). In these new experiments, we did stain the H3A31S-labeled fibers with PCNA and observed less asymmetric PCNA distribution (Figure 5D) compared to WT H3-labeled fibers (Figure 5C and 5A). We also added a Figure S4, where we show distribution of EdU pulse labeling on the replicative chromatin fibers derived from early-stage germ cells expressing WT H3 vs. mutant H3A31S. These data indicate that the EdU asymmetry is less on H3A31S-labeled chromatin fibers than on WT H3-labeled chromatin fibers.

References:

1. Xie J, Wooten M, Tran V, Chen BC, Pozmanter C, Simbolon C, et al. Histone H3 Threonine Phosphorylation Regulates Asymmetric Histone Inheritance in the Drosophila Male Germline. Cell. 2015;163(4):920-33. Epub 2015/11/03. doi: 10.1016/j.cell.2015.10.002

S0092-8674(15)01315-X [pii]. PubMed PMID: 26522592; PubMed Central PMCID: PMC4636931.
2. Alabert C, Barth TK, Reveron-Gomez N, Sidoli S, Schmidt A, Jensen ON, et al. Two distinct modes for propagation of histone PTMs across the cell cycle. Genes Dev. 2015;29(6):585-90. Epub 2015/03/21. doi: 10.1101/gad.256354.114. PubMed PMID: 25792596; PubMed Central PMCID: PMCPMC4378191.
3. Ma B, Trieu TJ, Cheng J, Zhou S, Tang Q, Xie J, et al. Differential Histone Distribution Patterns in Induced Asymmetrically Dividing Mouse Embryonic Stem Cells. Cell Rep. 2020;32(6):108003. Epub 2020/08/14. doi: 10.1016/j.celrep.2020.108003. PubMed PMID: 32783931; PubMed Central PMCID: PMCPMC7962874.
4. Petryk N, Dalby M, Wenger A, Stromme CB, Strandsby A, Andersson R, et al. MCM2 promotes symmetric inheritance of modified histones during DNA replication. Science. 2018;361(6409):1389-92. Epub 2018/08/18. doi: 10.1126/science.aau0294

science.aau0294 [pii]. PubMed PMID: 30115746.
5. Wooten M, Snedeker J, Nizami ZF, Yang X, Ranjan R, Urban E, et al. Asymmetric histone inheritance via strand-specific incorporation and biased replication fork movement. Nat Struct Mol Biol. 2019;26(8):732-43. Epub 2019/07/31. doi: 10.1038/s41594-019-0269-z

10.1038/s41594-019-0269-z [pii]. PubMed PMID: 31358945; PubMed Central PMCID: PMC6684448.
6. Wooten M, Li Y, Snedeker J, Nizami ZF, Gall JG, Chen X. Superresolution imaging of chromatin fibers to visualize epigenetic information on replicative DNA. Nat Protoc. 2020;15(3):1188-208. Epub 2020/02/14. doi: 10.1038/s41596-019-0283-y. PubMed PMID: 32051613; PubMed Central PMCID: PMCPMC7255620.
7. Kiger AA, Jones DL, Schulz C, Rogers MB, Fuller MT. Stem cell self-renewal specified by JAK-STAT activation in response to a support cell cue. Science. 2001;294(5551):2542-5. Epub 2001/12/26. doi: 10.1126/science.1066707

294/5551/2542 [pii]. PubMed PMID: 11752574.
8. Lim C, Gandhi S, Biniossek ML, Feng L, Schilling O, Urban S, et al. An Aminopeptidase in the Drosophila Testicular Niche Acts in Germline Stem Cell Maintenance and Spermatogonial Dedifferentiation. Cell Rep. 2015;13(2):315-25. Epub 2015/10/07. doi: 10.1016/j.celrep.2015.09.001

S2211-1247(15)01012-8 [pii]. PubMed PMID: 26440886; PubMed Central PMCID: PMC4607668.
9. Yamashita YM, Jones DL, Fuller MT. Orientation of asymmetric stem cell division by the APC tumor suppressor and centrosome. Science. 2003;301(5639):1547-50. Epub 2003/09/13. doi: 10.1126/science.1087795

301/5639/1547 [pii]. PubMed PMID: 12970569.

10. Tulina N, Matunis E. Control of stem cell self-renewal in Drosophila spermatogenesis by JAK-STAT signaling. Science. 2001;294(5551):2546-9. Epub 2001/12/26. doi: 10.1126/science.1066700. PubMed PMID: 11752575.
11. Boyle M, Wong C, Rocha M, Jones DL. Decline in self-renewal factors contributes to aging of the stem cell niche in the Drosophila testis. Cell Stem Cell. 2007;1(4):470-8. Epub 2008/03/29. doi: S1934-5909(07)00121-X [pii]

10.1016/j.stem.2007.08.002. PubMed PMID: 18371382.
12. Cheng J, Turkel N, Hemati N, Fuller MT, Hunt AJ, Yamashita YM. Centrosome misorientation reduces stem cell division during ageing. Nature. 2008;456(7222):599-604. Epub 2008/10/17. doi: 10.1038/nature07386. PubMed PMID: 18923395; PubMed Central PMCID: PMCPMC2712891.
13. Toledano H, D'Alterio C, Czech B, Levine E, Jones DL. The let-7-Imp axis regulates ageing of the Drosophila testis stem-cell niche. Nature. 2012;485(7400):605-10. Epub 2012/06/05. doi: 10.1038/nature11061

nature11061 [pii]. PubMed PMID: 22660319; PubMed Central PMCID: PMC4829122.
14. Wallenfang MR, Nayak R, DiNardo S. Dynamics of the male germline stem cell population during aging of Drosophila melanogaster. Aging Cell. 2006;5(4):297-304. Epub 2006/06/28. doi: ACE221 [pii]

10.1111/j.1474-9726.2006.00221.x. PubMed PMID: 16800845.
15. Ranjan R, Snedeker J, Chen X. Asymmetric Centromeres Differentially Coordinate with Mitotic Machinery to Ensure Biased Sister Chromatid Segregation in Germline Stem Cells. Cell Stem Cell. 2019;25(5):666-81 e5. Epub 2019/10/01. doi: S1934-5909(19)30349-2 [pii]

10.1016/j.stem.2019.08.014. PubMed PMID: 31564548.
16. Wooten M, Ranjan R, Chen X. Asymmetric Histone Inheritance in Asymmetrically Dividing Stem Cells. Trends Genet. 2020;36(1):30-43. Epub 2019/11/23. doi: 10.1016/j.tig.2019.10.004. PubMed PMID: 31753528; PubMed Central PMCID: PMCPMC6925335.
17. Ranjan R, Snedeker J, Wooten M, Chu C, Bracero S, Mouton T, et al. Differential condensation of sister chromatids acts with Cdc6 to ensure asynchronous S-phase entry in Drosophila male germline stem cell lineage. Dev Cell. 2022;57(9):1102-18 e7. Epub 2022/04/29. doi: 10.1016/j.devcel.2022.04.007. PubMed PMID: 35483360; PubMed Central PMCID: PMCPMC9134767.
18. Tran V, Lim C, Xie J, Chen X. Asymmetric division of Drosophila male germline stem cell shows asymmetric histone distribution. Science. 2012;338(6107):679-82. Epub 2012/11/03. doi: 10.1126/science.1226028

338/6107/679 [pii]. PubMed PMID: 23118191.
19. Xie J, Wooten M, Tran V, Chen X. Breaking Symmetry - Asymmetric Histone Inheritance in Stem Cells. Trends Cell Biol. 2017;27(7):527-40. Epub 2017/03/08. doi: S0962-8924(17)30025-9 [pii]

10.1016/j.tcb.2017.02.001. PubMed PMID: 28268050; PubMed Central PMCID: PMC5476491.
20. Ranjan R, Chen X. Quantitative imaging of chromatin inheritance using a dual-color histone in Drosophila germinal stem cells. STAR Protoc. 2022;3(4):101811. Epub 2022/11/18.

doi: 10.1016/j.xpro.2022.101811. PubMed PMID: 36386868; PubMed Central PMCID: PMCPMC9640340.
21. Lin S, Yuan ZF, Han Y, Marchione DM, Garcia BA. Preferential Phosphorylation on Old Histones during Early Mitosis in Human Cells. J Biol Chem. 2016;291(29):15342-57. Epub 2016/05/27. doi: 10.1074/jbc.M116.726067. PubMed PMID: 27226594; PubMed Central PMCID: PMCPMC4946945.
