# Supplementary material for: A single N-terminal amino acid determines the distinct roles of histones H3 and H3.3 in the Drosophila male germline stem cell lineage
Source: PLoS Biol. 2023 May 1;21(5):e3002098. doi: 10.1371/journal.pbio.3002098 (PMC10174566; doi:10.1371/journal.pbio.3002098)
Supplement: Attachment — Submitted filename: Responses to reviewers.pdf [file pbio.3002098.s026.pdf]

Reviewer #2: The manuscript has been improved with several new figures. Based on the information in authors' response, there are significant delay of lagging strand synthesis in GSCs, which authors plan to report in their future publication. If this is the case, newly formed histones (color switched to red) will of course distribute to lagging strand based on red protein availablity at the time of incorporation. However, old histones in original color does not necessarily "preexisting" histones. In this case, histone turn over time will be more important factor to understand distribution of each histone species (old vs new, preexisting vs newly formed).

We thank this reviewer for the positive feedback on our revision. We also thank this reviewer for the comment about using the tag switch method to distinguish old *versus* new histones, in particular for canonical histones whose incorporation mainly occurs during S phase. As we showed in Figure S1 and explained in our previous responses: The incorporation of new H3A31S is still replication-dependent, just like wild-type H3; and the incorporation of new H3.3S31A is still replication-independent, just like wild-type H3.3.

To clarify this point, we plan to add this discussion: "This tag switch method can differentially label any protein of interest in a spatiotemporally controlled manner. When applying to histones, the precise distinguishment between the two populations of histones (i.e., old *versus* new) is only applicable in the context of the actively ongoing cell cycle, and this precision declines over time. For example, the later expressed tagged histones are new during the first S phase but will become old during subsequent S phases. Contrastingly, the earlier expressed tagged histones represent old histones more precisely, but the turnover of these histones could cause this signal to diminish in following cell cycles. Additionally, as this tag switch occurs at the DNA level, it will take time for the switch to be reflected at the protein level, considering RNA stability and protein perdurance of old histone, as well as the time needed for the new histone gene to be transcribed, translated, and properly localized. Thus, this method is more appropriate for cell types with relatively long cell cycles such as *Drosophila* male GSCs, and it is important to monitor the production and incorporation of new histone in a time-course experiment to understand the dynamics of the tag switch at the protein level in the context of ongoing cell cycles."

This also affect the understanding of chic seq distribution data. In fact, endogenous histones are expressed specifically in S-phase, but UAS based expression timing must be continuous.

We agree with the expression of the transgenic histones outside S phase. However, as showed in Figure S1 and previous publication [1], the incorporation of newly synthesized canonical histone to the chromatin still mainly occurs during DNA replication. Further, the ChIC-seq utilized an unswitched, single GFP-tagged histones expressed under the germ cell-specific driver, in order to achieve cell-type-specific chromatin profiling using anti-GFP antibodies.

I would suggest to discuss potential reason and effect of asymmetric EdU strand observation and add more rigorous discussion about histone turnover timing.

In addition to more rigorous discussion about histone turnover timing as above, we added a discussion on potential reason and effect of asymmetric EdU strand: "The asymmetric EdU incorporation could reflect asynchronous synthesis between the leading strand and the lagging strand, which is likely compromised with H3A31A expression. Previously, we found that the

lagging strand-enriched component PCNA and lagging strand-specific protein RPA-70, a highly conserved single-stranded DNA-binding protein, both have asymmetric distribution on early-stage germ cell-derived chromatin fibers [2]. In contrast, such asymmetries of EdU and PCNA were much less observed in symmetrically dividing cultured Kc cells [3]."

Reference:

1. Kahney EW, Zion EH, Sohn L, Viets-Layng K, Johnston R, Chen X. Characterization of histone inheritance patterns in the Drosophila female germline. EMBO Rep. 2021:e51530. Epub 2021/05/26. doi: 10.15252/embr.202051530. PubMed PMID: 34031963.
2. Wooten M, Snedeker J, Nizami ZF, Yang X, Ranjan R, Urban E, et al. Asymmetric histone inheritance via strand-specific incorporation and biased replication fork movement. Nat Struct Mol Biol. 2019;26(8):732-43. Epub 2019/07/31. doi: 10.1038/s41594-019-0269-z 10.1038/s41594-019-0269-z [pii]. PubMed PMID: 31358945; PubMed Central PMCID: PMC6684448.
3. Wooten M, Li Y, Snedeker J, Nizami ZF, Gall JG, Chen X. Superresolution imaging of chromatin fibers to visualize epigenetic information on replicative DNA. Nat Protoc. 2020;15(3):1188-208. Epub 2020/02/14. doi: 10.1038/s41596-019-0283-y. PubMed PMID: 32051613; PubMed Central PMCID: PMCPMC7255620.
